# Study Protocol of the ESAP Study: Endoscopic Papillectomy vs. Surgical Ampullectomy vs. Pancreaticoduodenectomy for Ampullary Neoplasm—A Pancreas2000/EPC Study

**DOI:** 10.3389/fmed.2020.00152

**Published:** 2020-05-06

**Authors:** Marcus Hollenbach, Einas Abou Ali, Francesco Auriemma, Aiste Gulla, Christian Heise, Sara Regnér, Sébastien Gaujoux

**Affiliations:** ^1^Medical Department II—Gastroenterology, Hepatology, Infectious Diseases, Pulmonology, University of Leipzig Medical Center, Leipzig, Germany; ^2^Department of Gastroenterology, Digestive Oncology and Endoscopy, Cochin Hospital, Paris Descartes University, Paris, France; ^3^Digestive Endoscopy Unit, Division of Gastroenterology, Humanitas Clinical and Research Hospital, Rozzano, Italy; ^4^Institute of Clinical Medicine, Faculty of Medicine, Vilnius University, Vilnius, Lithuania; ^5^Center of Abdominal Surgery, Vilnius University Hospital Santaros Klinikos, Vilnius, Lithuania; ^6^Department of Surgery, Georgetown University University Hospital, Washington, DC, United States; ^7^Department of Medicine I—Gastroenterology, Pulmonology, Martin-Luther University Halle-Wittenberg, Halle, Germany; ^8^Section for Surgery, Department of Clinical Sciences Malmö, Lund University, Skane University Hospital, Malmö, Sweden; ^9^Department of Digestive, Hepatobiliary and Endocrine Surgery, Paris Descartes University, Cochin Hospital, Paris, France

**Keywords:** ampullectomy, ampulloma, pancreaticoduodenectomy, ampulla of vater, ERCP

## Abstract

**Background:** Lesions of the Ampulla of Vater are a rare condition and represent <10% of peri-ampullary neoplasms. Nevertheless, ampullary adenomas have the potential for malignant transformation to ampullary carcinomas by an adenoma-to-carcinoma sequence. Thus, adequate patient selection and complete resection (R0) of non-invasive ampullary lesions either by endoscopic papillectomy (EP), surgical ampullectomy (SA), or pancreaticoduodenectomy (PD) is essential. Although PD was traditionally performed, recent studies reported considerable efficacy and fewer complications following EP and SA. Since consistent comparative data are lacking, the Endoscopic Papillectomy vs. Surgical Ampullectomy vs. Pancreaticoduodectomy (ESAP) study will provide evidence for a therapeutic standard and post procedure morbidity in ampullary lesions.

**Methods:** International multicenter retrospective study. Adult patients (>18 years of age) who underwent SA or PD for ampullary neoplasm between 2004 and 2018 or EP between 2007 and 2018 will be evaluated. Main inclusion criteria are ampullary lesions strictly located to the ampulla. This includes adenoma, adenocarcinoma (T_1_ and T_2_), neuroendocrine tumors, gastrointestinal stroma tumors and other rare conditions. Exclusion criteria are peri-ampullary lesions, e.g., from the duodenal wall or the head of the pancreas, and interventions for tumor stages higher than T_2_. The main objective of this study is to analyze rates of complete resection (R0), recurrence and necessity for complementary interventions following EP, SA, and PD. Treatment-quality for each procedure will be defined by morbidity, mortality and complication rates and will be compared between EP, SA, and PD. Secondary objectives include outcome for patients with incomplete resection or initially understated tumors, lesions of the minor papilla, hereditary syndromes, neuroendocrine tumors, mesenchymal lesions, and other rare conditions. Additionally, we will analyze therapy by argon plasma coagulation and radiofrequency ablation. Furthermore, outcome in curative and palliative interventions can be distinguished.

**Conclusion:** The ESAP study will provide evidence for therapeutic algorithms and data for the implementation of guidelines in the treatment of different types of ampullary tumors, including recurrent, or incomplete resected lesions.

## Introduction

Lesions of the Ampulla of Vater are rare conditions. With a prevalence of less than 0.1%, they represent 7–10% of peri-ampullary lesions (1). Nevertheless, the rate of ampullary tumors has increased annually from 1973 to 2005 with a higher incidence in patients beyond the age of 50 (2). Ampullary tumors can be classified as benign, premalignant and malignant lesions (3). Thereby, histologic analysis reveal ampullary adenoma and adenocarcinoma in more than 90%, but also rare entities (e.g., neuroendocrine or mesenchymal lesions) have been described (4). As ampullary adenomas follow an adenoma-to-carcinoma sequence (5), they show a potential for malignant transformation (25–85%) and are considered as premalignant lesions (6). These lesions may also occur sporadically or can be linked to hereditary syndromes such as familial adenomatous polyposis (FAP). In patients with FAP, ampullary adenomas are very common and evolve in up to 80% with a 4% risk of malignant transformation (7).

Ampullary lesions usually present with non-specific symptoms and are often incidentally diagnosed on cross-sectional imaging or routine endoscopy. The most common presentation in symptomatic patients is painless jaundice (50–75%). Rare manifestations are cholangitis, acute pancreatitis as well as nausea, vomiting, biliary colic and weight loss (8).

Although the treatment of ampullary lesions is historically surgical, advances in endoscopic ultrasound (EUS) and endoscopic retrograde cholangio-pancreatography (ERCP) have significantly impacted the diagnostic and therapeutic procedures of patients with such a disease (9). Actually, ampullary lesions can be treated either by endoscopic ampullectomy or papillectomy (EP) (10), surgical or transduodenal ampullectomy (SA) (11) or pancreaticoduodenectomy (PD, pylorus-preserving pancreaticectomy or Whipple-Resection) (12). Despite clear consensus guidelines or recommendations are lacking, EP is currently mostly performed for smaller lesions (<20–50 mm) without any sign of invasive carcinoma, clear margins, soft tissue and absence of ulceration (13). However, the indications of EP are expanding. Recent studies describe the feasibility of “piece-meal” EP (14) even in large laterally spreading lesions (15), with deep ductal invasion (16) and supposed nodal-negative T_1_ adenocarcinoma (17). Additionally, EP can be used as a “macrobiopsy” for tumor staging, if the resection margins are compromised (18). This is important, as recent studies still show a limited pre-interventional accuracy of the endoscopic biopsy of 81.8% for ampullary adenoma (3.6% overseen malignancies) and only 66.7% for adenocarcinoma, despite of the use of EUS (19, 20).

To date, only a few studies compared EP and surgical techniques. These retrospective data revealed different inclusion criteria, outcomes, and surgical approaches. Nevertheless, a recently published meta-analysis of 5 studies summarized that surgery was more effective in ampullary adenoma, but was associated with higher rates of complications (21). However, this analysis showed several limitations. In particular, the reported complete resection rate after EP was dramatically lower than reported by the recent literature (>90%) (22).

In conclusion, the criteria to determine eligibility for endoscopic or surgical interventions in ampullary adenomas are not fully established and are far from a consensus. Thus, the Endoscopic Papillectomy vs. Surgical Ampullectomy vs. Pancreaticoduodectomy (ESAP) study will provide evidence for therapeutic algorithms of ampullary tumors, including recurrent or incomplete resected lesions and additional ablative therapies (23).

## Methods/Design

### Study Organization and Coordination

ESAP is designed and coordinated by the Pancreas2000/European Pancreatic Club study group. ESAP will be conducted as a retrospective multi-center study. The coordinating centers include the University of Leipzig Medical Center, Martin-Luther-University Halle-Wittenberg (Germany), Humanitas Clinical and Research Hospital (Italy), Lithuanian University of Health Sciences, Lund University (Sweden) and Cochin Hospital—Paris Descartes University (Paris, France). The investigators intend to include at least 40 participating centers. The study is investigator-initiated and receives no funding.

### Study Objectives

The primary objective of this study is to compare complete resection rates (R0-rate), determined by local pathologist, between EP, SA, and PD. Secondary aims include the rate of residual disease (defined as persistent lesion at the first endoscopic follow-up after the resection) and recurrence (defined as detectable lesion after initial negative follow-up). Additionally, disease-free and recurrence-free survival, length of hospital stay, 90-days post procedure complications and complementary interventions (argon plasma coagulation [APC], radiofrequency ablation [RFA], radiation, chemoradiotherapy and additional surgery) will be assessed. Furthermore, R0-rate, disease-free and recurrence-free survival and 90-days post procedure complications of ampullary lesions other than adenoma or adenocarcinoma (neuroendocrine tumors, gastrointestinal stroma tumors (GIST), mesenchymal tumors, paraganglioma, and hereditary polyposis syndromes) and lesions of the minor papilla will be examined.

### Patients, Inclusion and Exclusion Criteria

All adult patients (≥ 18 years of age), who underwent EP, SA, or PD for histologically proven ampullary lesions will be screened for eligibility for the study. As a follow-up of at least 12 months is required, patients in whom EP was performed between January 1st 2007 and July 31th 2018 can be included. For SA and PD, interventions can date back until January 1st 2004. The range of data to be analyzed was set different between endoscopic and surgical procedures, as endoscopic resection of ampullary lesions is a relatively new technique, and SA has been historically performed but is now a rare surgical procedure.

All histologic types of ampullary lesions should be included in this study. Regarding invasive ampullary carcinoma, only T_1_ and T_2_ M0 stage adenocarcinoma (UICC 8th edition) that were intended to treat can be included in this study.

Exclusion criteria are peri-ampullary lesions (duodenal tumor close to or involving the papilla, distal bile duct cancer invading the papilla and pancreatic adenocarcinoma) and ampullary adenocarcinoma higher than stage pT_2_ (UICC 8th edition) or with synchronous metastasis. In addition, data of patients with a follow-up of <12 months cannot be analyzed within this study. Inclusion and exclusion criteria for the study are listed in Table 1.

**Table 1:** Inclusion and exclusion criteria.

**Inclusion criteria**
Histologically proven ampullary lesion
Endocopic Papillectomy (EP), surgical ampullectomy (SA), pancreaticoduodenectomy (PD)
Intervention between January 1st 2007 (EP) or January 1st 2004 (SA and PD) and July 31th 2018
Age > 18 years
**Exclusion criteria**
Periampullary lesions
Ampullary adenocarcinoma higher than T_2_
Follow-up less than 12 months

### Study Design and Setting

ESAP is a retrospective, multicenter international study that aims to compare three different techniques for the therapy of ampullary tumors. As ampullary lesions are a rare condition, we try to include at least 40 participating centers with at least 10 complete data sets from each center.

Each site is required to have performed at least 10 interventions (EP, SA, or PD) for ampullary lesions in the indicated period. We are aware that this small case load could influence the results but we will stratify data for case load per center. Of course, the inclusion of both endoscopic and surgical patients is wanted but not mandatory. Endoscopy units and surgical theaters must meet all international quality standards and perform the interventions according to current technical recommendations.

In this study, detailed information regarding patients' medical history, performed interventional procedure, histology reports, and outcome are requested. In detail, age, gender, concomitant hereditary polyposis syndrome, anthropometrics, co-morbidities, medication, clinical presentation, and blood values will be assessed. Also, details of diagnostics including EUS, CT- and MRI-scan, prior interventions and intention to treat are necessary. Furthermore, the database will include information of endoscopic (stenting, sphincterotomy, submucosal injection, complementary treatment, lesion morphology) and surgical (duration, type of procedure and anastomosis, drains, margins, and complications) procedures. Histology reports will be screened for diagnosis of initial biopsy and resected specimen, size, R0-rate, deep and lateral margins, tumor stage, micro-/lymphovascular, and perineural invasions. Assessment of outcome includes length of hospital stay, mortality, residual and recurrent disease, additional treatment and long-term survival.

### Sample Size Considerations

The primary end point for the study is the rate of complete resection after the intervention, determined by clear margins in pathology. We are aware, that a considerable number of EP might be performed as “piece-meal” EP and thus is per definition not R0. Nevertheless, we also assess the rate of residual disease and recurrence of disease and these parameters will more precisely judge the impact of “piece-meal” resections. Recent published literature reported a success rate of EP between 46 and 92%. Thereby, the term “success” is inconsistently used and defined by R0-rate in some papers and complete endoscopic resection in others. In addition, an overall complication rate of 7.7% up to 42% (mostly minor complications) was reported (6). In contrast, own data from an ongoing meta-regression analysis (unpublished yet) indicated a *pooled mean* R0-rate for EP of 76.6%, for SA 96.4%, and 98.9% for PD. Nevertheless, data of the analyzed studies are heterogeneous and often difficult to compare. Thus, we estimated a conservative effect size of 0.22 with an alpha error of 5%. Simulations show a required sample size of 315 patients. As this is a retrospective analysis and equal distributions of patients between the EP, SA, and PD group as well as complete data sets cannot be guaranteed, we aim to include at least 400 patients to the final analysis.

### Statistical Analysis

The primary end point for the study is complete resection indicated by histology (R0-rate). To analyze R0-rate between the three groups, the 2 × 3 contingency table will be performed with a chi-squared test. Metric variables will be analyzed by ANOVA with Bonferroni-post-test. Depending on the dataset of the recruited patients, primary and secondary study objectives will also be analyzed by using a generalized linear mixed model (GLMM), which can take into account the longitudinal structure of the data as well as missing data. In addition, equal distribution between the three groups regarding baseline parameters (e.g., age, gender, co-morbidities, lesion size) may not be available. Thus, a propensity matched analysis is intended to overcome this possible limitation of the study.

Data will be presented as mean with standard deviation. Levels of significance should be presented by p-value and confidence interval. Odds ratios and absolute differences in proportions along with confidence intervals based on the logistic regression for the evaluation for predicting factors regarding primary and secondary objectives will be presented. Tests are all two-sided and the significance level is set at 5%. The final analysis will be performed after the last patient has included to the database.

### Ethical Considerations

The final study protocol was approved by the ethics committee of the Medical Faculty of the University of Leipzig (455/18-ek) in accordance with the declaration of Helsinki, the “Medical Association's Professional Code of Conduct” and the principles of ICH-GCP guidelines (issued in June 1996, ISO14155 from 2012). Furthermore, local legal and regulatory authorities as well as the medical secrecy and the Federal Data Protection Act will be followed. All participating centers also applied to their local ethics committees.

### Data Safety and Monitoring Board and On-Site Monitoring

The ESAP study is a multicenter retrospective study. Thus, the implementation of a data safety and monitoring board is not foreseen. Also, on-site monitoring is not necessary.

### Authorship

The first and last authorships are assigned to the ESAP coordinating authors. All collaborators will be cited either as author or contributor based on the number of data sets and the journal publication policy.

## Discussion

Ampullary lesions are a rare condition but its prevalence increased over the last decades (2). Particularly large lesions with indistinct margins are likely to undergo primary surgery. Nevertheless, indications for endoscopic resection are expanding, even in large laterally spreading tumors, and early stage adenocarcinoma (24). However, most published studies are monocentric with different inclusion criteria, patient characteristics, and measured outcomes. As a consequence of these heterogenic studies, the published rates of “treatment success” dramatically varies between 46 and 92% (22, 25–43). It is important to note that the term “treatment success” is inconsistently used. Often it is defined by R0-rate but also adopted for complete endoscopic resection or absence or recurrence and thus, can bias the results. Additionally, classifications and definition of complications are not uniform and range between 7.7 and 42% (mostly minor complications) but 30 day mortality was low between 0 and 1.9% (6).

In contrast, data of surgical ampullectomies are very few, and included between 11 and 44 patients per case series with an R0-rate between 63 and 100% (39, 44–57). Overall complications were between 9 and 68% but 30-day-mortality often was missing. Also, a lot of studies analyzed PD procedures over the last decades, but only a minority of them reported distinct outcomes for ampullary lesions. One could be impressed by the high R0-rates from 95.5% up to 100%, but these were reported by only 4 studies (49, 53, 58, 59) and included different patient populations. Also, overall complications range between 42.8 and 49.3% and perioperative mortality was not reported in this studies, but can be assumed significant (60, 61).

Our own data from an ongoing meta-regression analysis (unpublished data) indicated so far a *pooled mean* R0-rate for EA of 76.6%, for SA 96-4, and 98.9% for PD out of the current published literature. Unfortunately, these studies are heterogeneous and thus difficult to compare. This fact was also highlighted by a recent meta-analysis that aimed to compare endoscopic and surgical treatment, as both types of intervention together were rarely reported by only 4 studies (21). Although this work showed a higher rate of complete resection in the surgical group, this was accompanied by clearly more complications and this analysis was also limited by several inaccuracies. First, surgical procedures (SA and PD) were grouped, although these interventions are quite different with various short and long-term outcomes. Furthermore, a lot of papers, as mentioned before, could not be included, because this meta-analysis was restricted to studies presenting both types of interventions. In addition, there is an ongoing discussion if centers with small patients count are sophisticated enough to perform complex interventions such as endoscopic papillectomies or pancreticoduodenectomies. As we will include both centers with huge and small case load, we will stratify our data for this issue and hopefully will be able to give evidence-based recommendations for minimal requirements in the treatment of AL.

In conclusion, data regarding endoscopic or surgical therapy for ampullary tumors is heterogeneous and, at least in part, counterintuitive. Also, consensus guidelines or national/international recommendations are lacking. Therefore, the ESAP study will provide additional and robust data comparing EP, SA, and PD in ampullary adenoma and focal adenocarcinoma and will allocate evidence for therapeutic algorithms. Moreover, rarely addressed, but clinical important issues including recurrent or incomplete resected lesions, neuroendocrine and mesenchymal tumors, hereditary syndromes, and additional ablative therapy will be evaluated. In a consequence we plan to evaluate our results in a prospective validation study if we will be able to identify prediction parameters for primary and secondary outcomes.

## Ethics Statement

The studies involving human participants were reviewed and approved by Ethical Committee at the Medical Faculty, Leipzig University; IORG0001320, IRB00001750, chairwoman: Prof. Dr. Dr. Ortrun Riha, Käthe-Kollwitz-Str. 82, D-04109 Leipzig. Written informed consent for participation was not required for this study in accordance with the national legislation and the institutional requirements.

## Author Contributions

EA, MH, SG, SR, CH, FA, and AG: conception and design. MH, EA, FA, AG, and SG: literature search. MH, EA, CH, and FA: analysis of literature. MH: drafting the manuscript. SG, SR, CH, FA, EA, and AG: revising the manuscript. All authors agreed to be accountable for all aspects of the work in ensuring that questions related to the accuracy or integrity of any part of the work are appropriately investigated and resolved. All authors read and approved the final manuscript.

## Conflict of Interest

The authors declare that the research was conducted in the absence of any commercial or financial relationships that could be construed as a potential conflict of interest.

## Abbreviations

APC: Argon plasma coagulation
EP: Endoscopic papillectomy
ESAP: *E*ndoscopic Papillectomy vs. *S*urgical *A*mpullectomy vs. *P*ancreaticoduodectomy
FAP: Familial adenomatous polyposis
GIST: Gastrointestinal stroma tumor
PD: Pancreaticoduodenectomy
RFA: Radiofrequency ablation
SA: Surgical ampullectomy.
